# Migrated T lymphocytes into malignant pleural effusions: an indicator of good prognosis in lung adenocarcinoma patients

**DOI:** 10.1038/s41598-018-35840-3

**Published:** 2019-02-28

**Authors:** Juan C. Nieto, Carlos Zamora, José M. Porcel, Maria Mulet, Virginia Pajares, Ana M. Muñoz-Fernandez, Nuria Calvo, Iñigo Espinosa, Mónica Pascual-García, Silvia Bielsa, Silvia Vidal

**Affiliations:** 1 Department Immunology, Institut Recerca Hospital de La Santa Creu i Sant Pau, Barcelona, Spain; 2 Pleural Medicine Unit, Department Internal Medicine, Hospital Universitari Arnau de Vilanova, Lleida, Spain; 3 Department Pneumology, Hospital de la Santa Creu i Sant Pau, Barcelona, Spain; 4 Department Oncology, Hospital de la Santa Creu i Sant Pau, Barcelona, Spain; 5 Department Pathology, Hospital de la Santa Creu i Sant Pau, Barcelona, Spain

## Abstract

The presence of leukocyte subpopulations in malignant pleural effusions (MPEs) can have a different impact on tumor cell proliferation and vascular leakiness, their analysis can help to understand the metastatic microenvironment. We analyzed the relationship between the leukocyte subpopulation counts per ml of pleural fluid and the tumor cell count, molecular phenotype of lung adenocarcinoma (LAC), time from cancer diagnosis and previous oncologic therapy. We also evaluated the leukocyte composition of MPEs as a biomarker of prognosis. We determined CD4+ T, CD8+ T and CD20+ B cells, monocytes and neutrophils per ml in pleural effusions of 22 LAC and 10 heart failure (HF) patients by flow cytometry. Tumor cells were identified by morphology and CD326 expression. IFNγ, IL-10 and IL-17, and chemokines were determined by ELISAs and migratory response to pleural fluids by transwell assays. MPEs from LAC patients had more CD8+ T lymphocytes and a tendency to more CD4+ T and CD20+ B lymphocytes than HF-related fluids. However, no correlation was found between lymphocytes and tumor cells. In those MPEs which were detected >1 month from LAC diagnosis, there was a negative correlation between pleural tumor cells and CD8+ T lymphocytes. CXCL10 was responsible for the attraction of CD20+ B, CD4+ T and CD8+ T lymphocytes in malignant fluids. Concentrations of IL-17 were higher in MPEs than in HF-related effusions. Survival after MPE diagnosis correlated positively with CD4+ T and CD8+ T lymphocytes, but negatively with neutrophils and IL-17 levels. In conclusion, lymphocyte enrichment in MPEs from LAC patients is mostly due to local migration and increases patient survival.

## Introduction

The cellular content of pleural effusions differs depending on the etiology of the disease. Parapneumonic and malignant pleural effusions (MPE) are the most common causes of exudates, whereas heart failure (HF) causes the majority of transudates^1^. More than half of MPEs are due to metastases from lung and breast carcinomas^2^.

In patients with lung cancer, the presence of MPE contraindicates surgery and is predictive of poor prognosis, with a median survival of 5.5 months^3^. Pleural fluid formation in MPEs was traditionally considered to be the result of the tumorigenic obstruction of lymphatic vessels^4^. However, mouse models have revealed that MPE is the net product of an impaired pleural lymphatic drainage and increased fluid production due to extravasation from hyperpermeable pleural and/or tumor vessels induced by mediators that are released from tumor and tumor-recruited cells^5^. Autopsy studies have indicated that tumor cells metastasize to the pleural cavity mainly through the bloodstream^6^. Tumor-derived mediators directly stimulate inflammatory cell influx to the pleura and initiate vascular changes^7,8^. In the pleura, attracted leukocytes accumulate locally and have an impact on pleural tumor cell proliferation and vascular leakiness^9,10^.

The management of MPEs remains palliative due to its dismal prognosis^11^. Prognosis depends on several factors such as lactate dehydrogenase in pleural fluid, Easter Cooperative Oncology Group (ECOG) performance score, blood neutrophil-to-lymphocyte ratio, and primary tumor; all of which integrate to form a validated prognostic score in MPEs known as the LENT scoring system^12^. For the generation of the LENT score, the presence and type of pleural fluid leukocytes was not evaluated; an analysis which could be potentially valuable. Different types of comparisons have been established to analyze the leukocyte composition. When compared with peripheral blood, MPEs have more Th17, Th9 and Th1 lymphocytes^13,14^ and higher levels of IL-1 IL6, IL-17, and TGFβ^15,16^. Other authors described a bias towards a Th2 dominant state in MPEs from different primary neoplasms^17^. When compared with parapneumonic effusions, MPEs contain more lymphocytes, Tregs (regulatory T-cells), CD3+ and CD3 + CD25+ cells and less Th17^16^. Levels of IFN, IL-17, IL-16, and CCL20 were lower, but those of FoxP3, IL-10, TGFβ, and CCL17 were higher in MPEs than in tuberculous pleural effusions^16^. In fact, an elevated Treg/Th17 ratio in the MPEs of lung cancer patients is predictive of poor prognosis^17^. Divergences of these comparisons may be ascribed to variations in measurement techniques, sample preparations, failure to include control samples or methodological limitations^18^. Despite fluid cytology being a well-established diagnostic test for MPEs, its yield depends on sample preparation and cytologist experience and is insufficient for a detailed research analysis of leukocyte subsets^19^.

There is limited knowledge about the function of the leukocytes of MPEs. Macrophages from MPEs have a reduced cytotoxic activity and can inhibit tumor cell apoptosis^20^. Some reports have shown that T cell subsets are involved in sculping the pleural microenvironment that regulates intra-pleural tumor dissemination and pleural fluid accumulation^9^. In particular, CD4+ T lymphocytes contribute to immune evasion and facilitate tumor growth^21,22^, whereas CD8+ T lymphocytes have a defective cytotoxic potential^23,24^. In addition, abundant cytokines with immune-inhibitory properties have been described in pleural cavities affected by malignancy^25,26^.

Our main objective was to determine the lymphocyte subpopulation composition in the MPE of patients with lung adenocarcinoma (LAC). Since pleural effusions have cells in suspension, we were able to apply flow cytometry with multiple antibodies against specific markers to determine the cells per ml of each subpopulation. As controls, we included patients with effusions secondary to HF because they have a non-pathological representation of the local leukocyte populations^27^. To decipher the cause of MPE composition, we studied the association of leukocyte count per ml with the number of tumor cells and the clinical characteristics of the primary tumor (mutations, years of evolution, time from diagnosis, previous therapy). We also prospectively analyzed whether the leukocyte composition of this metastasic environment could be used as a surrogate biomarker for the prediction of survival in LAC patients. Overall, our findings provide new insights into the pathophysiological mechanisms of pleural metastases from lung cancer.

## Material and Methods

### Patients and sample collection

We analyzed 22 MPEs secondary to LAC and 10 benign effusions due to HF from patients who were attended at the Hospital Arnau de Vilanova (Lleida) and Hospital de la Santa Creu i Sant Pau (Barcelona). Samples were collected by thoracentesis and 10 U/ml of heparin were added to each sample. Diagnosis of MPE was based on positive cyto-histological studies. The study was approved by the Institutional Ethics Committee of the Hospital de la Santa Creu i Sant Pau. Patients signed an informed consent and samples were anonymized. Table 1 summarizes the patients’ clinical and pathological features, and the biochemistry of pleural fluids.

**Table 1:** Clinical characteristics of LAC and HF patients, and relative leukocyte composition of their pleural fluids.

	LAC	HF	P value
Patients, *n*	22	10	
Age, years^a^	64(60–74.73)	85(82.3–87.13)	n.s.
Male/female^b^	15/7	7/3	n.s.
Lactate Dehydrogenase (U/L)	614(410–927)	184(132–224)	<0.001
Total Protein (g/dL)	4.5(4.1–5.47)	2.9(2.4–3.4)	<0.001
Glucose (mg/dL)	104(85.2–169)	130(118–182)	n.s.
C-reactive protein (mg/L)	9.8(6.4–15.65)	5(2.2–14.5)	n.s.
Adenosine Deaminase (U/L)	9.3(2.9–22.9)	4.3(2.75–104)	n.s.
pH	7.4(7.34–7.44)	7.42(7.43–7.54)	n.s.
Mean (±SD) time from diagnosis, days	235 ± 444		
Mean (±SD) survival time from diagnosis, days	306 ± 218		
EGFR mutations	4/21		
ALK translocations	1/12		
**Treatment**			
Chemotherapy	6/22		
EGFR inhibitors	1/22		
Her2 inhibitors	1/22		
Immunotherapy	3/22		
Radiotherapy	1/22		
Surgery	1/22		
**Relative pleural fluid composition (%)** ^a^			
CD20+ B lymphocytes /lymphocytes	5.81(4.08–11)	3.71(0.75–6.01)	0.04
CD4+ T lymphocytes/lymphocytes	63(50.21–67.55)	63(52.08–73.78)	n.s.
CD8+ T lymphocytes/lymphocytes	23.52(17.86–29.97)	18.5(12.11–24.96)	n.s.
NK cells/lymphocytes	0.8(0.56–2.25)	2.94(1.3–9.51)	0.006
CD16 + CD14- neutrophils/leukocytes	0.78(0.26–2.8)	1.77(0.8–6.81)	n.s.
CD14+ monocytes/leukocytes	10.2(1.75–27.7)	23.9(10.71–42.26)	n.s.

ALK: anaplastic lymphoma kinase; EGFR: epidermal growth factor receptor; HF: heart failure; LAC: lung adenocarcinoma; MPE: malignant pleural effusion; NK: natural killer; n.s.: non-significant.

^a^Comparison by Mann Whitney test.

^b^Comparison by Fisher test.

### Staining and flow cytometry analysis of pleural fluid cells

Ten ml of pleural fluid were passed through a 40 µm filter and the cellular pellet was spun down. Then, red blood cells (RBC) were lysed using a soft RBC lysing solution (BioLegend, San Diego, CA). Cellular viability was then assessed using violet fluorescent reactive dye (Life Technologies, Carlsbad, CA). We included only samples with >95% cellular viability. The total number of nucleated cells was quantified in a MACSQuant cytometer (Miltenyi Biotec, Bergisch Gladbach, Germany). Cells were first incubated with FcR blocking and adjusted to 10 × 10^6^ cells/ml. One million cells were then stained with three panels of antibodies for 20 min at 4 °C. Panel A included anti-CD45-PE, CD20-FITC (Immunotools, Friesoythe, Germany), CD3-PECy5 and CD4-PECy7 or CD8-PECy7 (BioLegend). Panel B included anti-CD45-Vioblue (Miltenyi Biotec), CD14-APC, CD3-PECy5, CD16-FITC, and CD56-PE (Immunotools). Panel C included anti-CD45-Vioblue and CD326 (EpCAM)-PECy7 (Miltenyi Biotec). Cells were finally washed with PBS +1% BSA and resuspended in 300 µl for cytometry acquisition. For the analysis, aggregates, debris, and nonviable cells were excluded from our analysis based on forward (FSC) and side scatter (SSC). Leukocyte populations were identified based on the analysis of CD45+ cell gate. The least complex and smallest leukocytes corresponded to lymphocytes, and T and B subsets were identified as CD3+ and CD20+ cells, respectively. Inside the lymphocyte gate, NK cells were identified as CD3-CD16 + CD56 + cells, CD4+ T cells were analyzed as CD20− CD3 + CD4+, and CD8+ T cells as CD20− CD3 + CD8+. Monocytes and neutrophils were identified as CD14 + CD45+ and CD16 + CD14-CD45+ cells, respectively. Tumor cells were identified as CD45-CD326+ cells. The frequency (%) and cell number/ml were calculated for each cellular subset using the MACSQuantify software.

### Migration assay to malignant pleural fluid and analysis of migrated peripheral blood lymphocytes from healthy donors

Whole blood was lysed with RBC lysing solution (BioLegend). Cells were washed and the cell count was performed by MACSQuant cytometer. Cells were resuspended in RPMI 1640 with 10% fetal calf serum (Biological Industries, Kibbutz Beit Haemek, Israel), 2 mM glutamine, 100 U/ml penicillin, and 100 mg/ml streptomycin (Biowhittaker, Verviers, Belgium) at 2 × 10^6^ cells/ml. In 24 plate-wells, 500 μl of RPMI 1640 with 10% fetal calf serum or 0.22 μm filtered medium RPMI supplemented with 10% of pleural fluid from LAC was added to the wells. 3 × 10^5^ cells were added in 3 μm pore-size filters (Millipore Corporation, Billerica, MA) and incubated for 4 h at 37ΊC. After culture, healthy donor cells that migrated towards the medium or pleural fluids were collected from the wells, washed with PBS and stained with anti-CD3-PECy5, CD4-PECy7 (BioLegend) and CD20-FITC (Immunotools) monoclonal antibodies for 20 min. Cells were washed and resuspended in 300 μl of PBS to be acquired by flow cytometry. To analyze the migration fold of cells towards the pleural fluids, we calculated the relative cell migration that is the ratio between the absolute numbers of CD4+ T, CD8+ T cells and CD20+ B cells that migrate to the medium supplemented with pleural fluid and the absolute cell numbers of each respective subset that migrate to the medium.

### Proliferation assay of T cells from pleural effusions

To determine proliferation, 10^7^ T lymphocytes from LAC and HF fluids were labeled with 7 μM of 5(6)-carboxyfluorescein diacetate succinimidylester (CFSE) (Sigma, S. Louis, Missouri) for 15 minutes at 37 °C. The cells were then washed with pre-warmed medium, resuspended with complete medium at 5 × 10^6^ cells/ml and incubated with complete medium or with anti-CD3, anti-CD28, and anti-CD2 T cell activation/expansion kit for 72 hours according to the manufacturer’s instructions (Miltenyi Biotec). After culturing, pleural fluid cells were collected and stained with anti-CD3-PECy7, CD8-PErCP (BioLegend) and CD45-Vioblue and violet fluorescent reactive dye (Miltenyi Biotec). The cells were rewashed and resuspended in 300 μl of PBS.

### Determination of cytokine and chemokine concentrations in MPEs

Cell-free pleural fluids were obtained by centrifugation and then kept at −80 °C until use. IL-10 (Immunotools), IL-17 (Peprotech, London UK), IFNγ (BD Biosciences), MCP-1, CXCL4 and CXCL10 levels were determined using specific ELISA kits (Peprotech) according to the manufacturer’s instructions. All cytokines and chemokines were quantified with standard curves provided by the corresponding ELISA kit. The limits of detection were: 2 pg/ml for IL-10 and IL-17, and 30 pg/ml for IFNγ, CXCL10, CXCL4 and MCP-1.

### Statistical analysis

Statistical analyses were performed using Graph Pad Prism 7 software. Variables were reported as median (interquartile range –IQR-). The Kolmogorov-Smirnov test was applied to test for a normal distribution. Comparisons between groups were evaluated with the Mann Whitney test. Correlation analyses were carried out with the Spearman’s correlation test. P values < 0.05 were considered significant.

### Ethical approval

All procedures performed in studies involving human participants were in accordance with the ethical standards of the institutional and/or national research committee and with the 1964 Helsinki declaration and its later amendments or comparable ethical standards.

## Results

### Cellular composition of malignant and non-malignant pleural effusions

**Figure 1:**
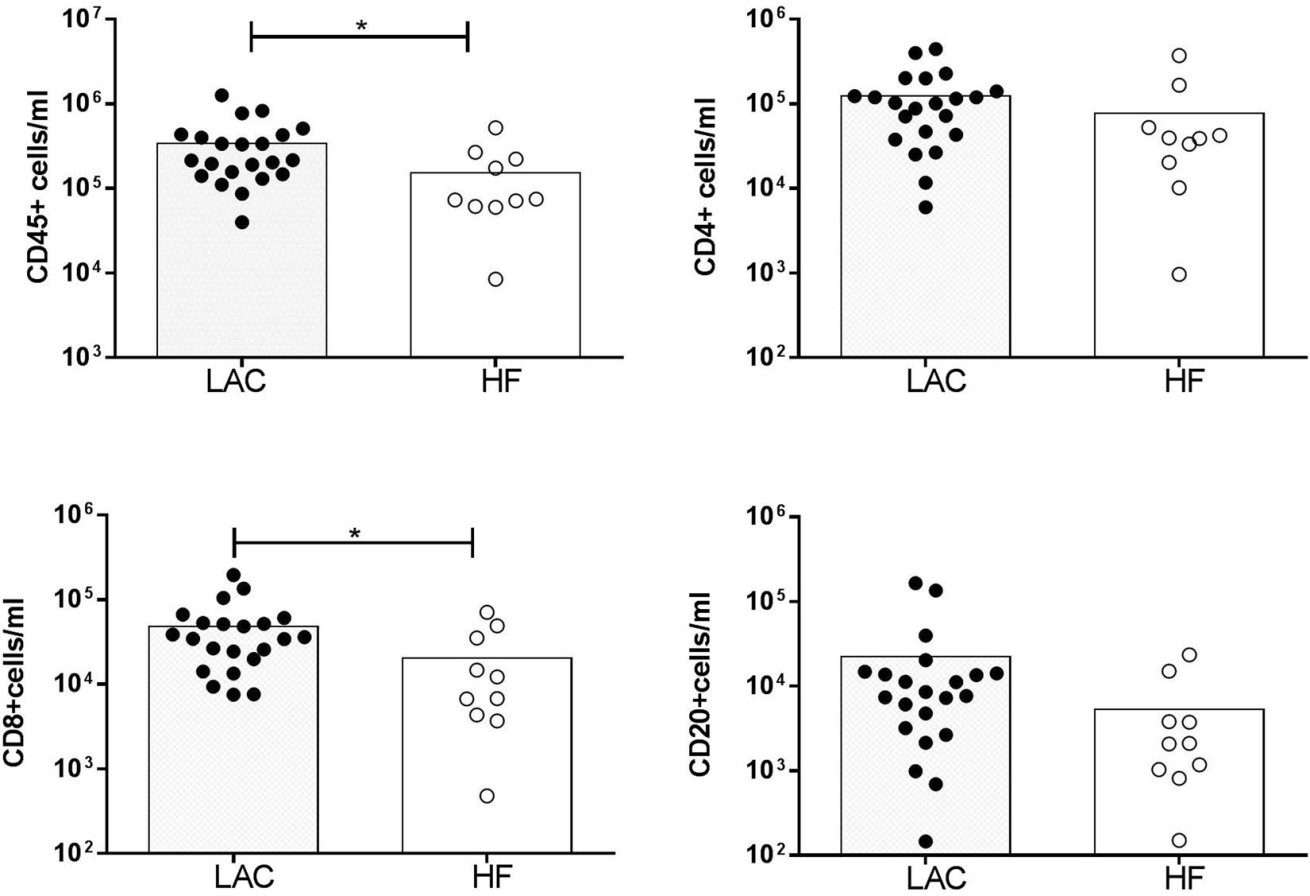
Leukocytes and lymphocyte subpopulations in malignant and non-malignant pleural effusions. Cells from MPEs of lung adenocarcinoma (LAC) and heart failure (HF) were stained with anti-CD3, CD4, CD14, CD16, CD20 and CD45 monoclonal antibodies to be analyzed by flow cytometry. Data are presented as mean. CD4+ cells/ml and CD20+ cells/ml in LAC and HF tended to be different (p = 0.04 and p = 0.06 respectively). Statistical analysis was performed using a non-parametric Mann-Whitney test. p < 0.05 was considered statistically significant. *p < 0.05.

There were more CD45+ cells per ml in MPEs from LAC than in pleural fluids from HF (Fig. 1). MPEs had more CD8+ T lymphocytes and a tendency to have more CD4+ T and B lymphocytes per ml than pleural fluids from HF patients. No differences in the NK cells, macrophages and neutrophils per ml were observed between LAC and HF effusions (NK: 2.65(1.30–6.33) × 10^3^ cells/ml for LAC vs 5.58(1.06–13.55) × 10^3^ cells/ml for HF; macrophages: 34.85(3.77–64.47) × 10^3^ cells/ml for LAC vs 16.09(7.31–46.82) × 10^3^ cells/ml for HF; neutrophils: 1.67(0.84–8.88) × 10^3^ cells/ml for LAC vs 1.28(0.61–9.94) × 10^3^ cells/ml for HF). The percentages of NK cells and CD20+ B lymphocytes, but not CD4+ T and CD8+ T lymphocytes, were different between LAC and HF pleural effusions (Table 1).

### Relationship between lymphocytes and tumor cells in malignant pleural effusions from lung adenocarcinoma

**Figure 2:**
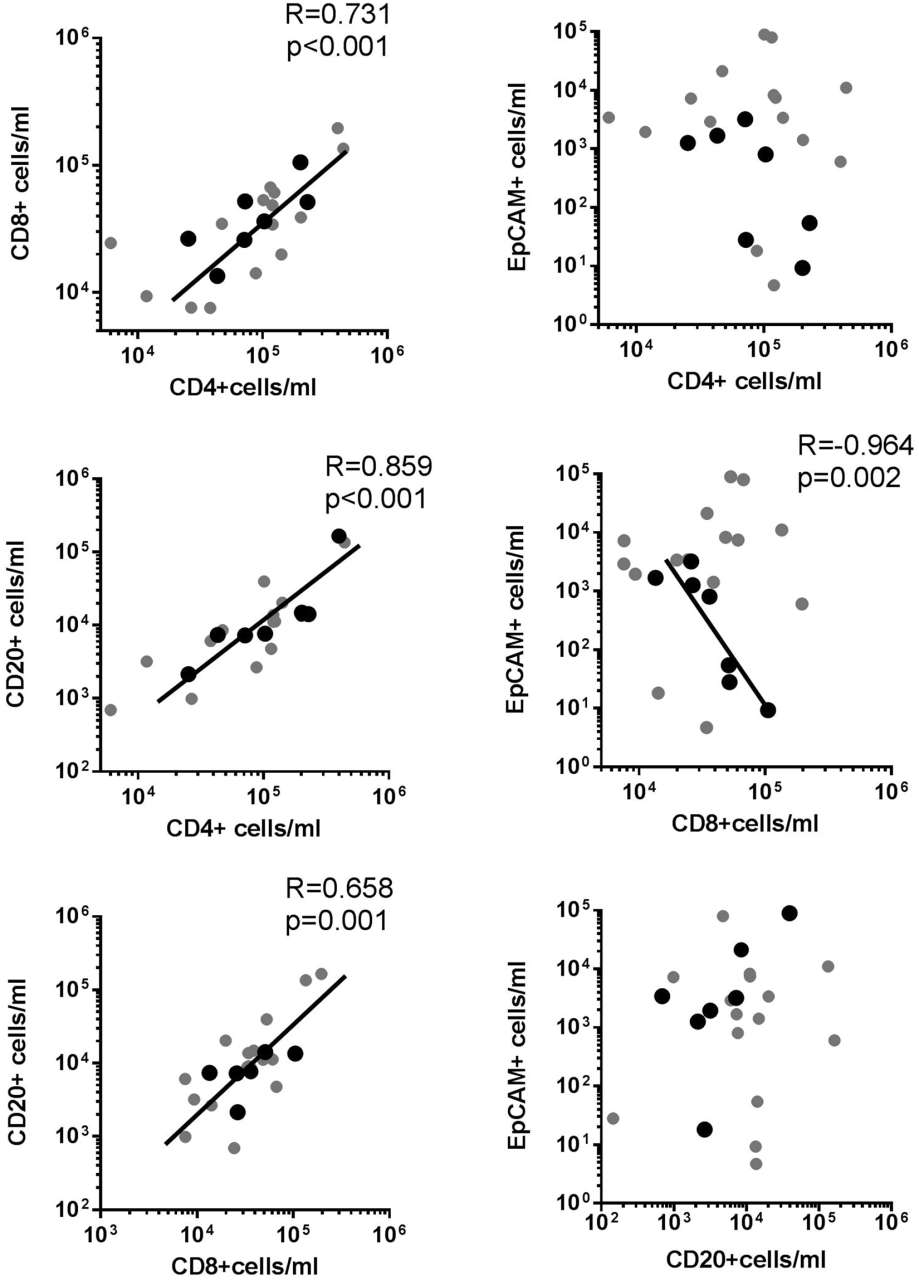
Correlation between lymphocyte populations and tumor cells in malignant pleural effusions from lung cancer. Cells from MPEs of LAC patients were stained with anti-CD3, CD4, CD20, CD45 and EpCAM (CD326) monoclonal antibodies to be analyzed by flow cytometry. Tumor cells were identified as CD45- CD326 (EpCAM)+ cells. Correlations between CD4+ T and CD8+ T, CD4+ T and CD20+ T, and CD8+ T and CD20+ B lymphocytes cells/ml and between tumor cells/ml and CD4+ T, CD8+ T and CD20+ B lymphocytes cells/ml were analyzed by Spearman’s correlation. Black circles correspond to LAC patients diagnosed with cancer >1 month before of MPE formation. Statistically significant correlations (p < 0.05) are shown.

A strong correlation between CD4+ T, CD8+ T and CD20+ B lymphocytes per ml was observed in MPEs from LAC (r = 0.731 for CD8+ vs CD4+ T, p < 0.001; r = 0.859 for CD20+ B vs CD4+ T, p < 0.001; and r = 0.658 for CD20+ B vs CD8+ T, p < 0.001) (Fig. 2). We next evaluated the correlation between the lymphocytes per ml of each subpopulation and the number of EpCAM+ CD45− tumor cells per ml. No correlation was observed between CD4+ T, CD8+ T or CD20+ B cells and tumor cells. However, selecting LAC patients that developed a MPE >1 month from the time of lung cancer diagnosis, a negative correlation between tumor cells and CD8+ T lymphocytes was observed (r = −0.964, p = 0.002). We did not find differences in any subpopulation of lymphocytes per ml between patients that had received treatment previous to the MPE development (n = 11) and those who did not (n = 11) (CD4+: 101.09(37.84–119.81) × 10^3^ cells/ml vs 102.96(43.09–202.55) × 10^3^ cells/ml; CD8+ T: 34.62(9.33–60.95) × 10^3^ cells/ml vs 36.20(24.45–52.03) × 10^3^ cells/ml; CD20+ B: 8.55(3.19–13.71) × 10^3^ cells/ml vs 7.65(2.13–14.88) × 10^3^ cells/ml, respectively). No differences in lymphocytes per ml were observed in patients with (n = 4) and without (n = 17) EGFR mutations (CD4+ T: 85.35(16.27–330.46) × 10^3^ cells/ml vs 101.09(40.46–130.42) × 10^3^ cells/ml; CD8+ T: 47.78(26.99–162.17) × 10^3^ cells/ml vs 34.23(13.81–51.68) × 10^3^ cells/ml; CD20+ B: 9.90(2.65–126.82) × 10^3^ cells/ml vs 7.37(2.92–13.97) × 10^3^ cells/ml, respectively). However, we found more EpCAM + CD45- tumor cells cells/ml in patients without previous oncologic treatment than in those that had received it (7.47(1.94–21.23) × 10^3^ cells/ml vs 1.26(0.05–3.22) × 10^3^ cells/ml, p = 0.02; respectively).

### Migration of lymphocytes to malignant pleural fluids and its association with CXCL10 concentration

**Figure 3:**
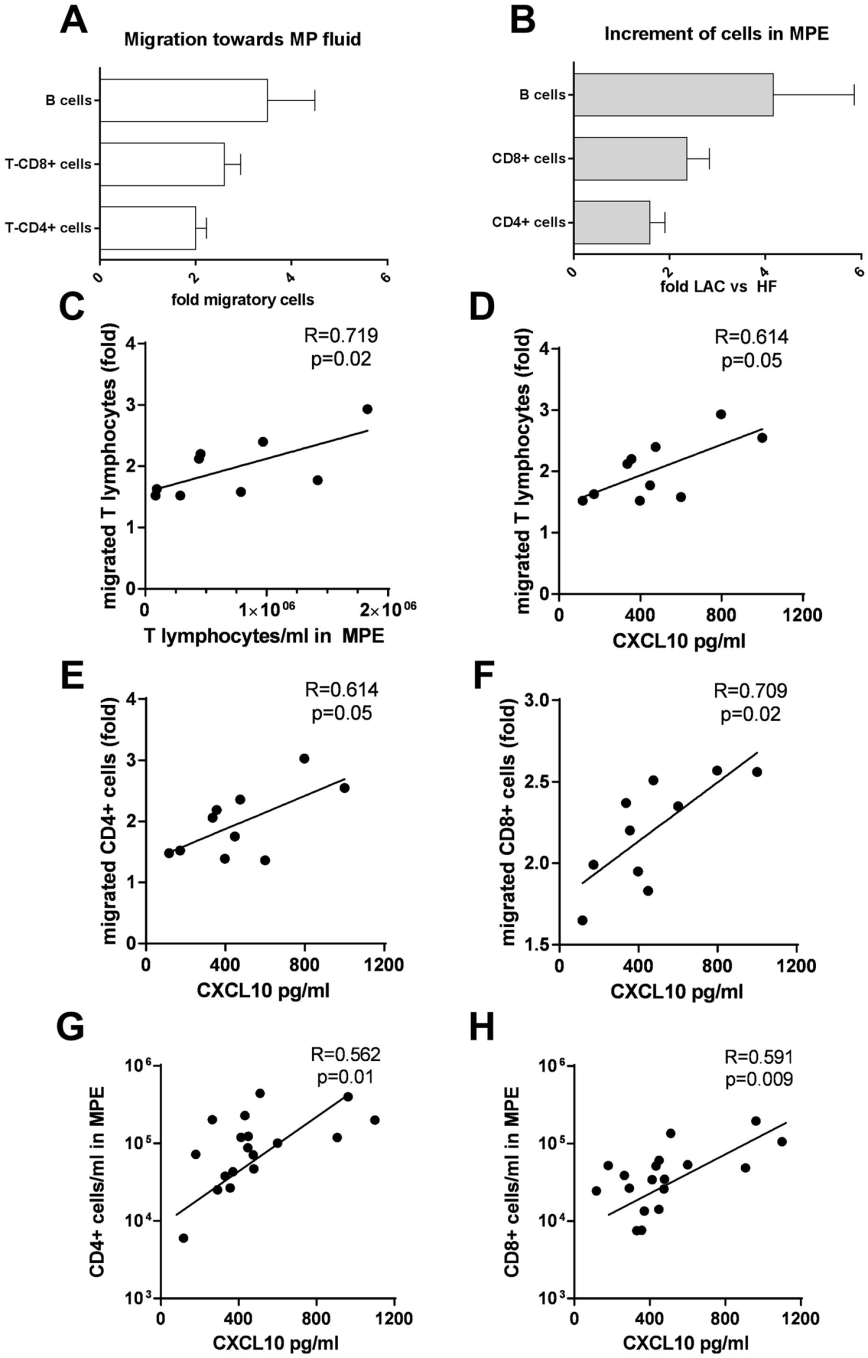
Association between migration of lymphocytes toward malignant pleural fluids and number of lymphocytes in malignant pleural effusions. Lysed whole blood cells from healthy donors (3 × 10^5^ cells) were added to 3 μm insert transwells. Under insert, culture medium with 10% of FCS or medium with 10% of MP fluid was added to the well. After 4 h, cells were collected from the well and stained with anti-CD3 and CD4. (**A**) Migration folds of CD20+ B, CD8+ T, and CD4+ T lymphocytes in a medium with 10% MP fluid vs medium with 10% FCS are shown. (**B**) Increments of CD20+ B, CD8+ T and CD4+ T lymphocytes in MPE from LAC vs HF are shown. Correlation of fold of migrated T lymphocytes with (**C**) T lymphocytes cells per ml in MPE and with (**D**) CXCL10 levels (pg/ml) in MP fluids. Correlation of CXCL10 levels with the fold of (**E**) migrated CD4+ T and (**F**) CD8+ T lymphocytes and (**G**) CD4+ T lymphocytes and (**H**) CD8+ T lymphocytes per ml in MPE. We used Spearman’s correlation. Statistically significant correlations (p < 0.05) are shown.

It is unlikely that the higher counts of lymphocytes per ml in MPEs as compared with non-MPEs could be explained by an increased cell proliferation in the former because we observed that CD4+ T and CD8+ T lymphocytes from both MPEs and non-MPEs had comparable spontaneous proliferation (data not shown). We then studied the capability of MPEs for attracting peripheral blood lymphocytes. It was observed that MPEs, attracted CD20+ B, CD4+ T and CD8+ T cells (relative cell migration towards MPE in fold change compared to control medium: CD20+ B: 2.85(1.65–5.12); CD8+ T: 2.35(2.05–3.07); CD4+ T: 1.95(1.47–2.5)) (Fig. 3). The relative cell migration of each lymphocyte subset to malignant pleural fluids was comparable to the increment of each subset in MPEs (increment of LAC cells in fold change compared to HF cells: CD20+ B: 1.51(0.57–2.68); CD8+ T: 1.73(0.9–2.69); CD4+ T: 1.31(0.53–2). In fact, relative cell migration towards malignant fluids and the increment of T lymphocytes per ml in MPEs strongly correlated (r = 0.719, p = 0.02). We next evaluated whether CXCL10 and MCP-1, two chemokines involved in T lymphocyte migration, could be playing a role in these findings. There was a correlation between the levels of total protein and CXCL10 (R = 0.717, p = 0.02). There was a correlation between CXCL10 levels (pg/ml) in MPEs and the CD4+ T and CD8+ T lymphocytes migrated to MPE (r = 0.614, p = 0.05; r = 0.46, p = 0.17 and r = 0.709, p = 0.02, respectively). In addition, the levels of CXCL10 in malignant fluids also correlated with the CD4+ T and CD8+ T cell count per ml (r = 0.562, p = 0.015 and r = 0.591, p = 0.009, respectively). No correlation was found between pleural fluid MCP-1 and CXCL4 levels and relative cell migration to malignant fluids and lymphocytes per ml in MPEs (data not shown).

### Levels of T-related cytokines in pleural fluids and their relationships with T lymphocyte populations

**Figure 4:**
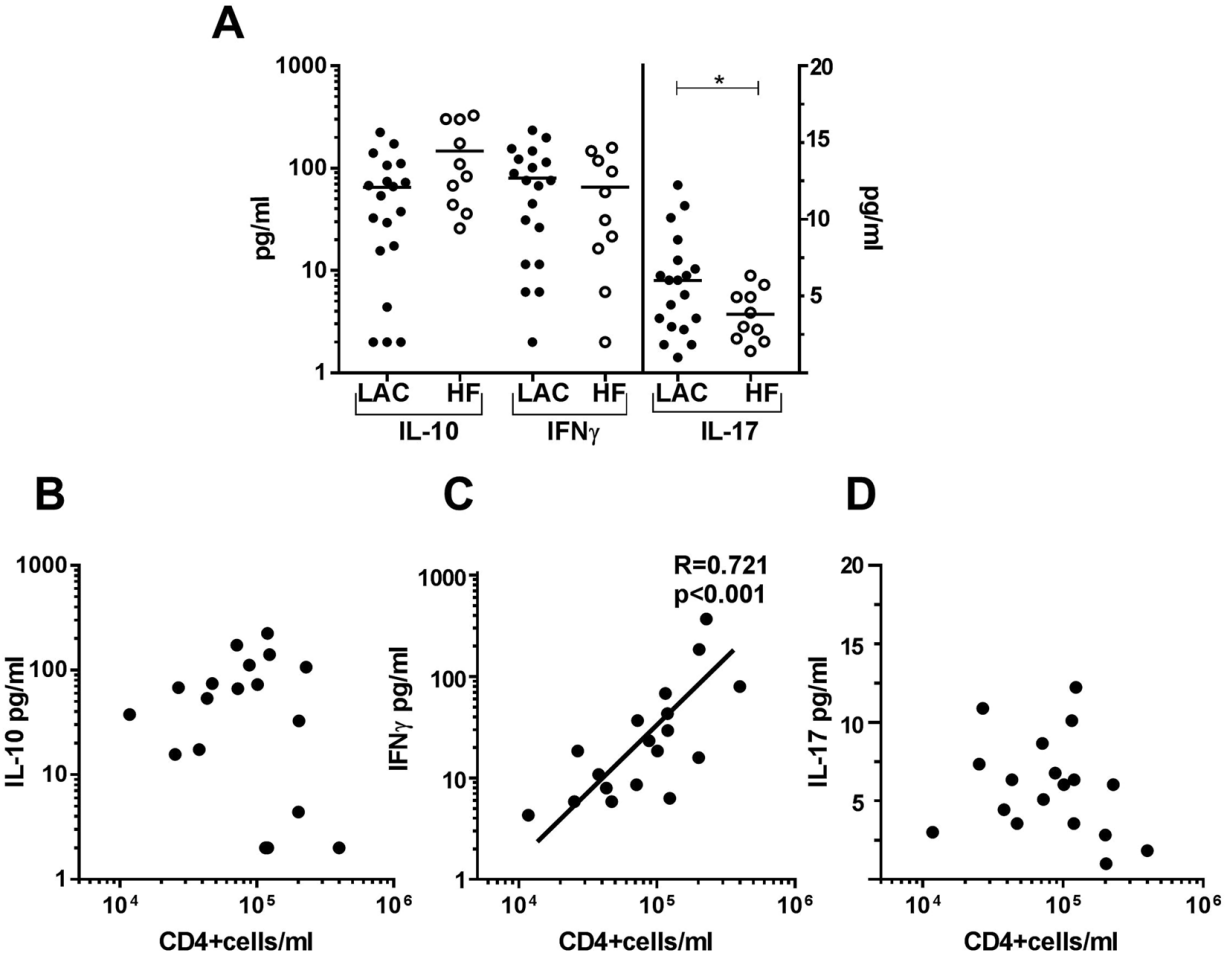
IL-10, IFNγ and IL-17 levels in MPE from LAC and pleural fluids from HF and the relationship of cytokines with T CD4+ lymphocytes per ml in MPE. Pleural fluids LAC and HF patients were filtered with 0.22 μm. (**A**) OD levels of IL-10, IFNγ, and IL-17 determined by ELISA. IL-10 levels in LAC and HF tended to be different (p = 0.06). Mann-Whitney test was used for comparison between LAC and HF. Correlation of CD4+ cells/ml with OD of (**B**) IL-10, (**C**) IFNγ and (**D**) IL-17 was analyzed by Spearman’s correlation. p < 0.05 was considered statistically significant. *p < 0.05.

We next compared the T-cell derived cytokines (IL-10, IFNγ, and IL-17) in malignant and benign pleural effusions. In the former, concentrations of IL-10 tended to be lower and those of IL-17 significantly higher than in the latter (Fig. 4). IFNγ levels in LAC and HF pleural fluids were comparable. There was a significant correlation between IFNγ levels (but not IL-10 and IL-17 levels) and the CD4+ T lymphocytes per ml in MPEs. No correlation was found between the levels of cytokines and the CD8+ lymphocytes per ml (data not shown).

### Association between leukocytes and cytokine levels in MPE and survival after MPE diagnosis

**Figure 5:**
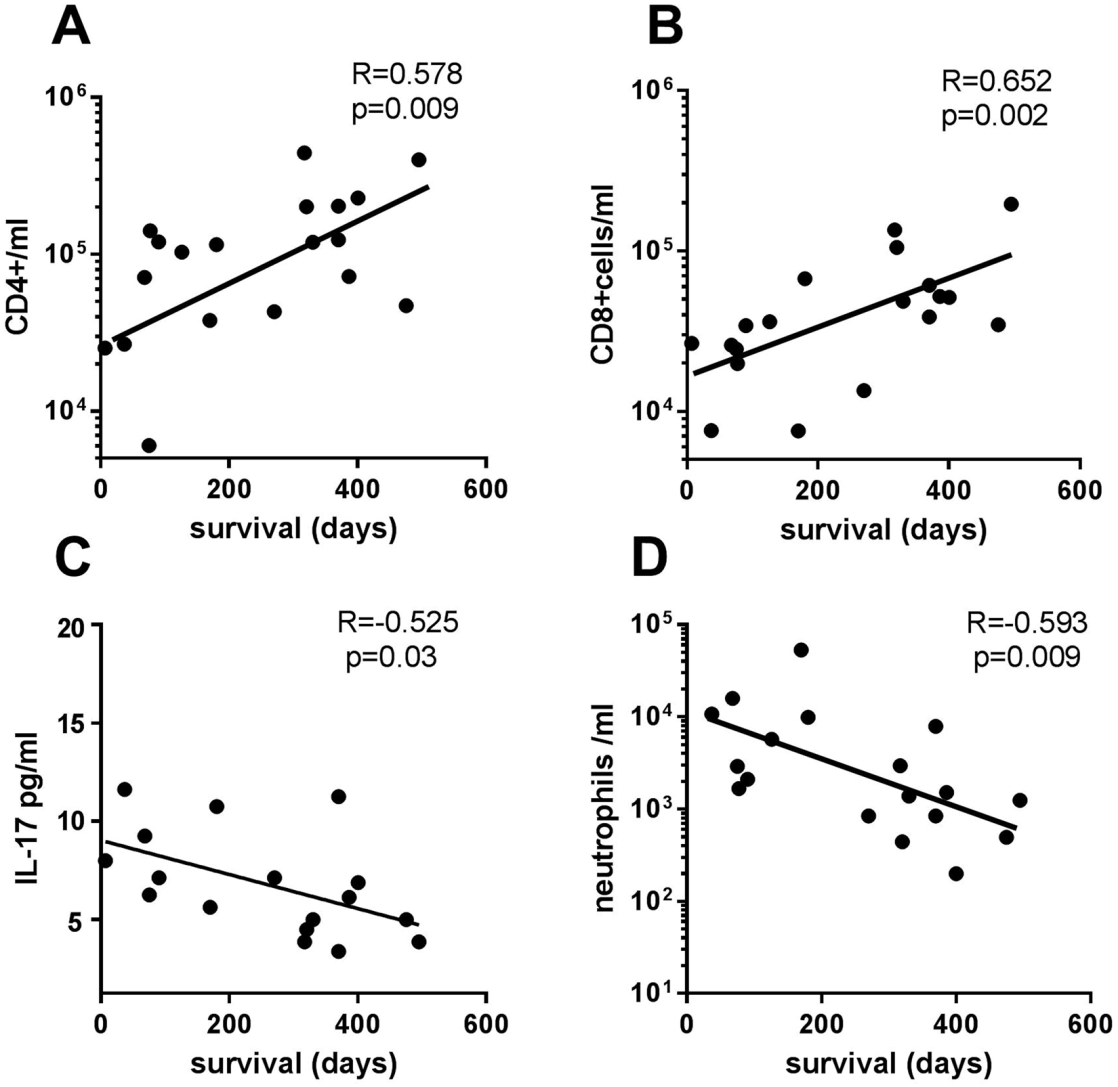
Association of cytokines and leukocytes per ml in MPE with survival days from MPE formation. Correlation between survival days after MPE formation and cells per ml of (**A**) CD4+ T, (**B**) CD8+ T, (**C**) IL-17 and (**D**) neutrophils per ml was analyzed by Spearman’s correlation. p < 0.05 was considered statistically significant.

We found that CD4+ T and CD8+ T lymphocytes per ml positively correlated with the days of survival after MPE diagnosis (r = 0.578, p = 0.009 and r = 0.652, p = 0.002, respectively) (Fig. 5). CD20+ B lymphocytes also tended to correlate with survival (r = 0.454, p = 0.051, data not shown). Neutrophils per ml and the levels of IL-17, but not of IL-10 and IFNγ, negatively correlated with survival from MPE diagnosis (r = −0.525, p = 0.03, and r = −0.593, p = 0.009, respectively). In fact, the levels of IL-17 and neutrophils per ml were positively correlated (r = 0.485, p = 0.03; data not shown).

## Discussion

Lymphocytes are more abundant in MPEs from LAC than in HF-related effusions. There was a correlation between the absolute counts of CD4+ T, CD8+ T and CD20+ B lymphocytes per ml in MPEs. This finding suggests that the increase of each subset originated from a common mechanism. Using an *in vitro* experiment we showed that each subpopulation of lymphocytes was attracted to malignant fluids, and CXCL10 was a crucial chemokine involved in this phenomenon. The survival of LAC patients was positively correlated with the presence of CD4+ T and CD8+ T lymphocytes but negatively correlated with the presence of neutrophils and the levels of IL-17 in the pleural fluid.

It was shown that there were more lymphocytes, but not monocytes or neutrophils, in MPE than in benign fluids. Also, lymphocytes were mostly CD4+ and the ratio CD4+/CD8+ was similar in both malignant and non-malignant pleural effusions^28,29^. Our findings of lymphocytes being the predominant cell population in MPEs are comparable with some previous studies^30^. However, other reports analyzing the percentages instead of cell counts have suggested a different conclusion. For instance, Leiser *et al*. reported a lower accumulation of CD8+ T cells but a higher percentage of CD14+, dendritic and mesothelial cells in MPEs compared to benign pleural effusions. In contrast to our study, benign pleural effusions were represented by inflammatory diseases where the inflammatory mediators could be different^17^ Scherpereel *et al*. also reported that pleural fluids from donors with essential hyperhidrosis, considered as controls because of the absence of thoracic disease, contained mainly CD8+ T cells, while MPEs included mostly CD4+ T lymphocytes^24^. There are two major differences that could explain the discrepancy between control group findings when either heart failure or essential hyperhidrosis is used. One difference is that effusions from hyperhidrosis were obtained by pleural lavage (injection and immediate aspiration of 150 mL of prewarmed saline into the pleural space) whereas heart failure effusions, as MPE, were obtained by direct thoracentesis. Another possible difference is that the previous therapies in heart failure and hyperhidrosis could have distinctively altered the leukocyte repertoire of these effusions. Prado-Garcia *et al*. showed that in MPEs from primary lung cancer, the percentage of CD8+ T cells, in particular, the terminally differentiated subset, was reduced compared to non-malignant effusions because they underwent antibody-induced cell death^31^. Two possible differences between these reports and our findings could be that they compared lymphocyte percentages and that malignant fluids might have been collected at a different time from diagnosis. This last aspect could be relevant because the predominant leukocyte subpopulation could depend on the phase of the pleural metastasis^32^. The latter, however, seems unlikely because we were not able to correlate the time from diagnosis to lymphocyte count per ml in our cohort of patients. To conclusively evaluate the possibility of changes in the predominant cell population, the collection of consecutive samples from the same patient will be of interest in future studies.

Although an increased influx of circulating monocytes and macrophage differentiation is expected during an inflammatory response associated with cancer, macrophage counts did not increase in MPEs and we, similarly to Risberg *et al*., could not establish any relationship between the presence of macrophages and CD326+ tumor cells^33^. Immunophenotyping of macrophage subpopulations may further reveal details of particular macrophages and their relationship with tumor cells.

We agree with other reports in that neutrophils constitute <25% of the cell population in MPEs^34^. However, we found an increase in neutrophils in those MPEs with more tumor cells, suggesting their recruitment from circulating blood through the local release of chemotactic factors. Consistently, some reports have shown a significant positive correlation between pleural IL-8 concentrations and neutrophil counts^35^.

Our findings suggest that the increased number of lymphocytes in MPE were due to migration rather than proliferation, which is in agreement with reports showing a defective proliferative capacity of MPE lymphocytes. Yang *et al*. have previously demonstrated that CD4+ T lymphocytes frequently accumulate in metastatic MPEs^16^. However, we did not find a positive correlation between the number of tumor cells and lymphocytes.

Migration of leukocytes has been a crucial mechanism in experimental MPE because CCL2 and anti-CCL12 blockades were able to inhibit MPE development in mouse models^36^. Lymphocyte migration can also be supported by the higher levels of osteopontin, SSP1, and VEGF in exudates, which promote vascular hyperpermeability and correlate with pleural inflammation^5^. In our cohort of patients, we have found that lymphocytes migrated towards malignant fluids in the same proportion that they were present in pleural fluids. The *in vitro* migration of T lymphocytes and the lymphocyte count per ml in MPEs were positively correlated with the pleural concentration of CXCL10. Although we cannot rule out the involvement of other CXCR3 ligands, our findings suggest a relevant role for CXCL10, linked to the attraction of Th1, in the accumulation of lymphocytes in MPEs. Although we did not find a significant increase of IFNγ in malignant fluids, its consumption cannot be ruled out in this setting. The complex kinetics of cytokines and lymphocytes require further studies about the production of IFNγ and the role of other chemokines at different times from the diagnosis of LAC and after therapy.

A higher concentration of IL-17 in MPEs than in HF-related effusions was found, and the IL-17 levels were inversely correlated with survival. It has already been reported that there are higher IL-17 levels in MPEs than in non-malignant effusions, with values below 15 pg/ml being associated with longer overall survival^37^. The role of Th17, the main T cell subset that produces IL-17, in cancer is hardly conclusive. Controversially, Th17 cells have been found in both pro- and antitumorigenic processes^38,39^. The protumor function mediated by Th17 and IL-17 has been demonstrated both in animal tumor models and patients with cancer^40,41^, and a higher ratio Treg/Th17 was highly correlated to poor survival^16^. IL-17 can act as an angiogenic factor accelerating tumor growth and metastasis through neo-vascularisation. Th17 is also able to induce the secretion of inflammatory cytokines such as IL-8 and TNF which will attract neutrophils and destroy the niche of immunity^42,43^. On the other hand, Th17 may contribute to protective tumor immunity via stimulating the production of Th1 chemokines CXCL9 and CXCL10 to recruit effector cells to tumor tissues^44^ and to elicit the activation of tumor-specific CD8+ T cells^45^. The number of Th17 cells has been reported to be elevated in MPEs, a fact which predicted the improved survival of these patients. Ye *et al*. also showed that the number of Th17 was significantly increased in the MPEs of patients with prolonged survival, implying a beneficial role^43^. However, our findings are in accordance with the traditionally reported role of Th1 cells and IFN as the major mediators of antitumor immunity.

Despite being focused on the immunological microenvironment of pleural metastasis, this study was limited to LAC patients. It is likely that MPEs from patients with other malignancies have other immunological patterns due to differences in time of diagnosis, evolution, therapy and tumor characteristics.

Using flow cytometry, we were able to establish a quantitative comparison of lymphocyte subpopulations between pleural effusions related to LAC and HF. It was found that migration has a central role in the predominance of pleural fluid lymphocytes and that IL-17 levels and attracted neutrophils are indicative of poor prognosis. These findings suggest that MPE cells are a convenient source to study the host-tumor interactions. Due to the inherent disseminated stage of MPE secondary to LAC, this could also be an excellent model to study the evasion mechanisms of tumors. Accurate characterization of tumor cells and tumor-associated leukocytes by flow cytometry may help to introduce an innovative diagnostic tool and open new prospects for cell therapy in MPEs. However, we cannot ignore that these pleural tumor cells are metastatic and that cells from the primary tumor could have a different phenotype and response.

### Compliance with Ethical Standards

Research Involving Human Participants. We obtained signed informed consent.
